# Comparison of mixed-model approaches for association mapping in rapeseed, potato, sugar beet, maize, and Arabidopsis

**DOI:** 10.1186/1471-2164-10-94

**Published:** 2009-02-27

**Authors:** Benjamin Stich, Albrecht E Melchinger

**Affiliations:** 1 Department of Applied Genetics and Plant Breeding, Institute of Plant Breeding, Seed Science and Population Genetics, University of Hohenheim, 70593, Stuttgart, Germany

## Abstract

**Background:**

In recent years, several attempts have been made in plant genetics to detect QTL by using association mapping methods. The objectives of this study were to (i) evaluate various methods for association mapping in five plant species and (ii) for three traits in each of the plant species compare the *T*_*opt*_, the restricted maximum likelihood (REML) estimate of the conditional probability that two genotypes carry at the same locus alleles that are identical in state but not identical by descent. In order to compare the association mapping methods based on scenarios with realistic estimates of population structure and familial relatedness, we analyzed phenotypic and genotypic data of rapeseed, potato, sugar beet, maize, and Arabidopsis. For the same reason, QTL effects were simulated on top of the observed phenotypic values when examining the adjusted power for QTL detection.

**Results:**

The correlation between the *T*_*opt *_values identified using REML deviance profiles and profiles of the mean of squared difference between observed and expected *P *values was 0.83.

**Conclusion:**

The mixed-model association mapping approaches using a kinship matrix, which was based on *T*_*opt*_, were more appropriate for association mapping than the recently proposed QK method with respect to the adherence to the nominal *α *level and the adjusted power for QTL detection. Furthermore, we showed that *T*_*opt *_differs considerably among the five plant species but only marginally among different traits.

## Background

Artificially induced variations, such as mutations, have been successfully used for gene identification in genetic and physiological studies [1]. Development of DNA markers, however, has made it possible to study the naturally occuring allelic variation underlying complex traits [2,3]. In many plant species, the approaches for detecting quantitative trait loci (QTL) relied so far on segregating populations derived from crosses between inbred lines. These QTL detection procedures, commonly referred to as linkage mapping, have major limitations, that include high costs [4] and a poor resolution in detecting QTL. Moreover, with biparental crosses of inbred lines only two alleles at any given locus can be studied simultaneously [5]. Association mapping methods, which have been successfully applied in human genetics to detect genes coding for human diseases [6], promise to overcome these limitations [7]. Therefore, in recent years several attempts have been made in a plant genetics context to detect QTL by using such methods [7-10].

Linkage disequilibrium (LD) in linkage mapping populations is caused by genetic linkage [9]. In contrast, LD in association mapping populations can also be the consequence of population structure, relatedness, genetic drift, and selection [5,11]. Therefore, the success of association mapping efforts depends on the ability to separate LD due to linkage from LD due to other causes. To correct for LD caused by population structure, linear models accounting for sub-population effects [8] or a logistic regression ratio test [12,13] were proposed. Owing to the large germplasm sets required for dissecting complex traits [14], the probability of including partially related individuals increases. This applies in particular when genotypes selected from plant breeding populations are used for association mapping [7,9,13]. However, the above-mentioned approaches fail to adhere to the nominal *α *level if the germplasm set analyzed comprises related individuals [13].

The recently proposed QK mixed-model for association mapping promises to correct for LD caused by population structure and familial relatedness [15]. The authors demonstrated the suitability of their new method for association mapping in two allogamous species, humans and maize. The suitability of the QK method, however, has to be evaluated in plant species with different reproduction systems covering a wide range of population structure and familial relatedness.

In contrast to coancestry coefficients calculated from pedigree records, marker-based kinship estimates may account for the effects of deviations from expected parental contributions to progeny due to selection or genetic drift [16]. Therefore, marker-based kinship estimates might be more appropriate for association mapping approaches than coancestry coefficients calculated from pedigree records [15,17]. A difficulty with calculation of marker-based kinship estimates is the definition of unrelated individuals [18]. The marker-based kinship matrix might be determined based on the definition that random pairs of genotypes are unrelated [15] or that pairs of genotypes are unrelated if they have no allele in common [17]. However, both definitions seem to be arbitrary. Recently, it was proposed to estimate by restricted maximum likelihood (REML) *T*_*opt*_, the conditional probability that marker alleles are alike in state, given that they are not identical by descent [19], using genotypic and phenotypic data [20]. However, no study compared this estimation of unrelated individuals among plant species with different reproduction systems as well as for various phenotypic traits.

The objectives of our study were to evaluate various methods for association mapping with respect to their adherence to the nominal *α *level and the adjusted power for QTL detection based on (i) empirical data sets and (ii) computer simulations in five plant species with different reproduction systems. Second, we compared *T*_*opt *_for three traits in each of the plant species.

## Methods

With computer simulations it is hardly possible to simulate data sets showing a population structure and familial relatedness comparable to that of empirical data sets. Nevertheless, to compare association mapping methods with respect to their adherence to the nominal a level based on scenarios with realistic estimates of population structure and familial relatedness, we analyzed phenotypic and genotypic data of rapeseed, potato, sugar beet, maize, and Arabidopsis. For the same reason, QTL effects were simulated on top of the observed phenotypic values when examining the adjusted power for QTL detection.

### Plant materials, phenotypic data, and molecular markers

In each of the five plant species, with the exception of *Arabidopsis thaliana*, we selected three traits with different genetic complexity (presumably low, medium, and high). Detailed descriptions of the examined data sets are available as Additional file 1.

#### Rapeseed (*Brassica napus *L.)

We studied a total of *n *= 136 rapeseed inbreds, proprietary to Norddeutsche Pflanzenzucht Hans-Georg Lembke KG (Holt-see, Germany). The entries were evaluated for thousand kernel weight (TKW; g), oil content (OC; %), and oil yield (OY; t/ha). All entries were fingerprinted with *m *= 59 genome-wide distributed simple sequence repeat markers by Saaten-Union Resistenzlabor GmbH (Hovedissen, Germany) following standard protocols.

#### Potato (*Solanum tuberosum *L.)

Our study was based on the phenotypic and genotypic data evaluated earlier [21]. Briefly, the *n *= 184 tetraploid potato clones from the breeding programs of Böhm-Nordkartoffel Agrarproduktion OHG (Lüneburg, Germany) and Saka-Ragis Pflanzenzucht GbR (Windeby, Germany) were evaluated for *Globodera pallida *St. resistance (GPR) [22]. Our statistical analyses were based on the square root of the number of visible nematode cysts. Furthermore, the area under the disease progress curve [23] was used as measure for *P. infestans *resistance (PIR). In addition, plant maturity (PM) was evaluated in uninfected plants, using a 1 to 9 scale (1 = very early, 9 = very late). All entries were fingerprinted with *m *= 31 genome-wide distributed simple sequence repeat markers [21] by the potato genome analysis group of the Max Planck Institute for Plant Breeding Research (Cologne, Germany). For 21 markers the allele dosage was scored based on relative band intensities.

#### Sugar beet (*Beta vulgaris *L.)

We analyzed a total of *n *= 178 sugar beet inbreds of the pollen parent heterotic pool, proprietary to KWS SAAT AG (Einbeck, Germany). The test-cross progenies of these entries with an inbred of the seed parent heterotic pool were evaluated in a series of plant breeding trials. Data were recorded for amino nitrogen (AN) [24], beet yield (BY), and corrected sugar yield (CSY) [25] in % of the mean performance of checks. All entries were fingerprinted with 59 simple sequence repeat markers and 41 single nucleotide polymorphism markers (*m *= 100), both randomly distributed across the sugar beet genome. The fingerprinting was done by KWS SAAT AG following standard protocols.

#### Maize (*Zea mays *L.)

Our study was based on the phenotypic and genotypic data analyzed earlier [15]. In short, the *n *= 277 maize inbreds representing worldwide genetic diversity were evaluated for ear height (EH; cm), ear diameter (ED; cm), and days to pollen shed (DPS). For all inbreds, genotypic data of *m *= 553 genome-wide distributed single nucleotide polymorphism markers was available.

#### *Arabidopsis thaliana *L.

Our study was based on the *n *= 95 *Arabidopsis thaliana *L. inbreds for which phenotypic information was available [17]. These inbreds represent world-wide genetic diversity of Arabidopsis. We examined the normalized gene expression of *FLOWERING LOCUS C *(FLC) and *FRIGIDA *(FRI) as well as the number of days from germination to first opening of flowers under long day conditions with vernalisation treatment (LDV). For these inbreds, resequencing data of *m *= 876 genome-wide distributed short fragments was available [26]. To reduce the computational load, we used only the central single nucleotide polymorphism marker of each fragment.

The anonymised data sets of rapeseed, potato, and sugar beet are available upon request from the authors.

### Statistical analyses

The empirical type I error rate of association-mapping approaches based on adjusted entry means (two-step approaches) is only slightly higher than that of approaches in which the phenotypic data analysis and the association analysis were performed in one step (one-step approaches) [20]. Therefore, in a first step we analyzed the phenotypic data and calculated adjusted entry means (rapeseed, potato) or entry means (sugar beet, maize, and Arabidopsis) *M*_*i *_for each individual under consideration (Additional file 2). These estimates were then used in a second step for the association analyses.

#### Association analyses

For each of the five plant species, nine different statistical models (Table 1), which were described in detail previously [20], were used to calculate the *P *value for the association of each of the *m *marker loci with each of the three phenotypic traits. The entries of four of the five plant species in our study were homozygous inbred lines (Table 2) and, thus, no inferences can be made about dominance effects. Furthermore, for potato, di-, tri, and tetragenic effects [27] were neglected in our study.

**Table 1:** Methods used for association mapping and the corresponding statistical models.

Method	Statistical model	Population structure matrix **D**	Kinship matrix **K**
ANOVA	$M_{i}=\mu +\alpha^{\prime }X_{i}+e_{i}$	-	-
K	$M_{i}=\mu +\alpha^{\prime }X_{i}+g_{i}^{*}+e_{i}$	-	SPAGeDi
Q_1_K	$M_{i}=\mu +\alpha^{\prime }X_{i}+\sum_{u=1}^{z}D_{iu}v_{u}+g_{i}^{*}+e_{i}$	STRUCTURE; Δ*K *criterion	SPAGeDi
Q_2_K	$M_{i}=\mu +\alpha^{\prime }X_{i}+\sum_{u=1}^{z}D_{iu}v_{u}+g_{i}^{*}+e_{i}$	STRUCTURE; Log likelihood	SPAGeDi
PK	$M_{i}=\mu +\alpha^{\prime }X_{i}+\sum_{u=1}^{z}D_{iu}v_{u}+g_{i}^{*}+e_{i}$	Principal components; explaining simultaneously 25% of the variance	SPAGeDi
K_*T*_	$M_{i}=\mu +\alpha^{\prime }X_{i}+g_{i}^{*}+e_{i}$	-	$K_{Tij}=\frac{S_{ij}-1}{1-T}+1$; *T *= 0,0.025, ..., 0.975
Q_1_K_*T*_	$M_{i}=\mu +\alpha^{\prime }X_{i}+\sum_{u=1}^{z}D_{iu}v_{u}+g_{i}^{*}+e_{i}$	STRUCTURE; Δ*K *criterion	*T *= 0,0.025, ..., 0.975
Q_2_K_*T*_	$M_{i}=\mu +\alpha^{\prime }X_{i}+\sum_{u=1}^{z}D_{iu}v_{u}+g_{i}^{*}+e_{i}$	STRUCTURE; Log likelihood	*T *= 0,0.025, ..., 0.975
PK_*T*_	$M_{i}=\mu +\alpha^{\prime }X_{i}+\sum_{u=1}^{z}D_{iu}v_{u}+g_{i}^{*}+e_{i}$	Principal components; explaining simultaneously 25% of the variance	*T *= 0,0.025, ..., 0.975

For a detailed definition of the statistical models and description of the different methods see Materials and Methods.

**Table 2:** Description of the examined data sets.

Parameter	Rapeseed	Potato	Sugar beet	Maize	Arabidopsis
*n*	136	184	178	277	95
Entry type	Inbred line	Non-inbred clone	Inbred line	Inbred line	Inbred line
*Phenotypic data*					
Trait 1	Thousand kernel weight	Resistance to *G. pallida*	Amino nitrogen	Ear height	Norm. gene expression of *FLC*
Abbrev.	TKW	GPR	AN	EH	FLC
Unit	g	$\sqrt{\text{No}\text{. of nematode cysts}}$	%	cm	%
*h*^2^	0.78	0.98	0.89	-	-
Range *M*_*i*_	3.0–4.6	0.4–19.5	71.1–226.2	8–136	0.021–6.270
Trait 2	Oil content	Resistance to *P. infestans*	Beet yield	Ear diamter	Norm. gene expression of *FRI*
Abbrev.	OC	PIR	BY	ED	FRI
Unit	%	Area under disease progress curve	%	mm	%
*h*^2^	0.81	0.77	0.90	-	-
Range *M*_*i*_	46.1–51.7	-6.4–165.1	84.8–113.6	23.7–46.4	0.211–4.386
Trait 3	Oil yield	Plant maturity	Corrected sugar yield	Days to pollen shed	Flowering time
Abbrev.	OY	PM	CSY	DPS	LDV
Unit	t/ha	Rating scale 1 to 9	%	No. of days	No. of days
*h*^2^	0.50	0.94	0.81	-	-
Range *M*_*i*_	2.2–3.0	3.4–9.5	87.8–108.7	54.5–82.5	18.7–55.7
*Genotypic data*					
Type of markers	SSRs	SSRs	SSRs & SNPs	SNPs	SNPs
*m*	59	31	100	553	876
Avg. allele freq.	0.37	0.18	0.30	0.50	0.50

*M*_*i *_is the adjusted entry mean (rapeseed and potato) or entry mean (sugar beet, maize, and Arabidopsis) of the *i*th genotype calculated over all environments.

The first model was an ANOVA model of the form:

$$M_{i}=\mu +\alpha^{\prime }X_{i}+e_{i}$$

where ***α ***were the effects of allele substitution of the marker under study, **x**_*i *_a column vector with the number of copies of the corresponding alleles, and *e*_*i *_the residual.

The statistical model underlying our mixed-model association mapping approaches was:

$$M_{i}=\mu +\alpha^{\prime }x_{i}+\sum_{u=1}^{z}D_{iu}v_{u}+g_{i}^{*}+e_{i},$$

where *v*_*u *_was the effect of the *u*th column of the population structure matrix **D **and $g_{i}^{*}$ was the residual genetic effect of the *i*th entry. The matrix **D**, which comprised *z *linear independent columns, differed among the examined mixed-model association mapping methods (Table 1), which is why it is described in the paragraphs on the individual methods. The variances of the random effects g* = $\left\{g_{1}^{*},...,g_{n}^{*}\right\}$ and **e **= {*e*_1_, ..., *e*_*n*_} were assumed to be Var(g*) = $2K\sigma_{g^{*}}^{2}$ and Var(**e**) = $R\sigma_{r}^{2}$, where **K **was a *n *× *n *matrix of kinship coefficients that define the degree of genetic covariance between all pairs of entries. $\sigma_{g^{*}}^{2}$ was the residual genetic variance and $\sigma_{r}^{2}$ the residual variance, both estimated by REML. **R **was an *n *× *n *matrix in which the off-diagonal elements were 0 and the diagonal elements were reciprocals of the number of phenotypic observations underlying each entry mean or adjusted entry mean [15].

The K method was based on the above described mixed-model with the difference that it did not include any *v*_*u *_effects (Table 1). The kinship matrix **K **was calculated based on all marker data using the software package SPAGeDi [28], where negative kinship values between entries were set to 0.

The Q_1_K and Q_2_K methods were based on the above described mixed-model. For these two methods, the population structure matrices **Q**_1 _and **Q**_2_, which were calculated using software STRUCTURE [29] and described in the following paragraphs, were used as **D **matrix. In our investigations, the set of *n *entries was analyzed by setting *z *from 0 to 14 in each of five repetitions. For each run of STRUCTURE, the burn-in time as well as the iteration number for the Markov Chain Monte Carlo algorithm were set to 100 000 [30].

For the **Q**_1 _matrix, the number of sub-populations was estimated based on the ad-hoc criterion Δ*K *[31]. In contrast, for the **Q**_2 _matrix, we used the run with the highest log likelihood to and the lowest number of sub-populations [32]. The *z *+ 1 columns of both, the **Q**_1 _and **Q**_2 _matrix, add up to one and, thus, only the first *z *columns were used as **D **matrix of the Q_1_K and Q_2_K method, respectively, to achieve linear independence. The Q_1_K and Q_2_K methods were based on the same kinship matrix **K **as used for the K method.

We used the first *p *principal components of an allele frequency matrix as **D **matrix of the PK method (Table 1) [17]. *p *was chosen in such a way that the explained variance of the first *p *principal components was about 25%. The PK method was based on the same kinship matrix **K **as used for the K method.

The Q_1_K_*T*_, Q_2_K_*T*_, PK_*T*_, and K_*T *_methods were based on a matrix **K**_**T **_which was calculated according to:

$$K_{Tij}=max\left(1-\left(\frac{1-S_{ij}}{1-T}\right),0\right),$$

where *S*_*ij *_is the proportion of marker loci with shared variants between inbreds *i *and *j *[20]. We examined *T *= 0, 0.025, ..., 0.975 to obtain a REML estimate of *T*, which is the conditional probability that marker alleles are alike in state, given that they are not identical by descent.

#### Measures for comparison of association mapping methods

The mean squared difference (MSD) between observed and expected *P *values of all marker loci was calculated as measure for the adherence to the nominal *α *level [20]. High MSD values indicate that the empirical type I error rate of these approaches is considerably higher than the nominal *α *level. Computer simulations were performed based on a bivariate beta-distribution [33] to examine which difference in MSD values between two association mapping methods could be expected purely by chance [20]. For each trait of each plant species, we investigated five pairs of association mapping approaches (i) Q_1_K/ANOVA, (ii) Q_1_K/K, (iii) Q_1_K/Q_2_K, (iv) Q_1_K/PK, and (v) Q_1_K/$Q_{1}{\text{K}}_{T_{opt}}$.

For each of the five plant species, the Pearson correlation coefficient between the observed *P *values of all association mapping methods was calculated for the trait with medium genetic complexity.

#### Power simulations

The power to detect a biallelic QTL of interest, which explained a fraction of the phenotypic variance and was in complete LD with one marker locus, was examined as described in detail previously [20]. Briefly, the QTL effect *G*_*r*_, calculated as *r *= 0.1 multiplied by the standard deviation of the vector of adjusted entry means **m **= (*M*_1_, *M*_*i*_, ..., *M*_*n*_) of the *n *entries, was assigned in consecutive simulation runs to each of the detected marker alleles whereas all other alleles were assigned the genotypic effect 0. In each simulation run, the phenotypic value of each entry *i *was calculated by summing up the QTL effect of the alleles and the adjusted entry mean *M*_*i*_. All association mapping methods were run on the phenotypic values of the entries to determine whether the QTL can be detected. To adjust the association mapping methods for their different empirical type I error rates, we calculated the adjusted power as the proportion of QTL detected, based on the nominal *α *for which the empirical type I error rate α* was 0.05. In addition to *r *= 0.1, we examined *r *= 0.4, 0.7, ..., 1.9. The percentage (*π*) of the total phenotypic variation explained by a QTL effect *G*_*r *_was calculated [15].

All mixed-model calculations were performed with ASReml release 2.0 [34].

## Results

For each trait examined in the current study, considerable variation was observed for the entry means or adjusted entry means *M*_*i *_(Table 2). The total number of marker alleles detected for rapeseed, potato, sugar beet, maize, and Arabidopsis was 331, 158, 176, 1106, and 1752, respectively. The average allele frequency ranged from 0.18 for potato to 0.50 for maize and Arabidopsis.

The model-based approach of STRUCTURE revealed *z *+ 1 = two, two, two, five, and six sub-populations for rapeseed, potato, sugar beet, maize, and Arabidopsis, respectively, when using the ad-hoc criterion Δ*K*. In contrast, based on SBC, the number of sub-populations revealed by STRUCTURE was 11, 15, 10, 15, and 5. For rapeseed, potato, sugar beet, maize, and Arabidopsis, the minimum number of principal components *p *explaining simultaneously 25% of the variance was 4, 5, 4, 13, and 8, respectively.

The MSD between observed and expected *P *values of the K approach ranged from 0.0002 (maize, ED) to 0.0604 (potato, PM) and was considerably lower than that of the ANOVA approach ranging from 0.0004 (Arabidopsis, FRI) to 0.1928 (potato, GPR) (Table 3). For the Q_1_K and Q_2_K methods, the MSD values were of similar size and varied between 0.0002 (maize, DPS) and 0.0389 (potato, PM). The MSD value of the PK method ranged from 0.0002 (maize, DPS) to 0.0422 (potato, PM).

**Table 3:** Mean of squared differences (MSD) between observed and expected *P *values for various association mapping methods in five plant species.

Method	Rapeseed		
	
	TKW	OC	OY
ANOVA	0.0624	0.0326	0.0523
K	0.0098	0.0053	0.0016
Q_1_K	0.0021	0.0047	0.0061
Q_2_K	0.0013	0.0010	0.0192
PK	0.0008	0.0007	0.0026
	Potato		
	
	GPR	PIR	PM
ANOVA	0.1928	0.0947	0.1534
K	0.0499	0.0162	0.0604
Q_1_K	0.0122	0.0179	0.0389
Q_2_K	0.0181	0.0017	0.0063
PK	0.0189	0.0183	0.0422
	Sugar beet		
	
	AN	BY	CSY
ANOVA	0.1526	0.1625	0.1533
K	0.0136	0.0191	0.0173
Q_1_K	0.0060	0.0239	0.0051
Q_2_K	0.0118	0.0167	0.0081
PK	0.0090	0.0137	0.0065
	Maize		
	
	EH	ED	DPS
ANOVA	0.0333	0.0147	0.0909
K	0.0003	0.0002	0.0006
Q_1_K	0.0002	0.0003	0.0002
Q_2_K	0.0002	0.0002	0.0003
PK	0.0003	0.0005	0.0002
	Arabidopsis		
	
	FLC	FRI	LDV
ANOVA	0.0040	0.0004	0.0070
K	0.0006	0.0022	0.0013
Q_1_K	0.0026	0.0033	0.0017
Q_2_K	0.0019	0.0022	0.0013
PK	0.0021	0.0034	0.0018

For abbreviations of the analyzed traits see Table 2. For a detailed definition of the statistical models and description of the different methods see Materials and Methods.

**Figure 1:**
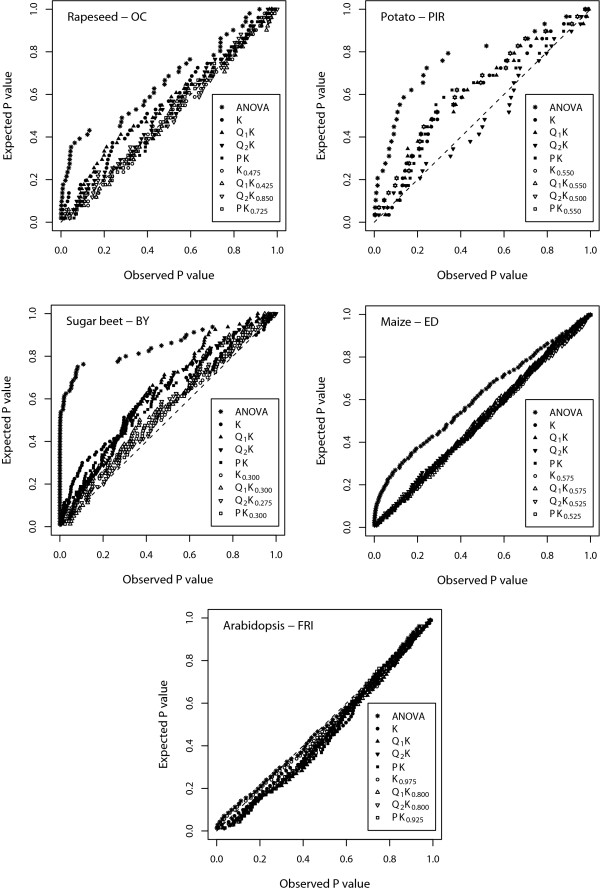
**Plot of observed *vs*. expected *P *values for the nine different association mapping methods**. For maize, every fifth, and for Arabidopsis, every eigth *P *value was plotted to increase the clarity of the plot. For each of the five plant species, the result of the trait with medium genetic complexity is presented.

For all plant species, traits, and mixed-model approaches examined, considerably different values of REML-based deviance as well as MSD were observed for the examined levels of *T *(Additional file 3). The optimum threshold *T*_*opt*_, identified based on deviance profiles, ranged from 0.450 to 0.925 (Table 4). By comparison, the threshold *T*_*opt*_, identified based on MSD profiles, ranged from 0.275 to 0.975. The correlation between the *T*_*opt *_values identified using these two criteria was 0.83 (Additional file 4). The MSD values observed for the mixed- model approaches, which were based on the $K_{T_{opt}}$ matrix, were lower than that observed for the approaches which were based on the **K **matrix (Table 3; Table 4; Fig. 1).

**Table 4:** *T *values for which the lowest deviance or the lowest mean of squared differences between observed and expected *P *values were found for various association mapping methods in five plant species.

Mixed-model method	deviance	MSD	deviance	MSD	deviance	MSD
	Rapeseed					
	
	TKW		OC		OY	
			
K_*T*_	0.725	0.350 (0.0068)	0.800	0.475 (0.0006)	0.750	0.400 (0.0011)
Q_1_K_*T*_	0.775	0.700 (0.0039)	0.800	0.425 (0.0007)	0.775	0.700 (0.0011)
Q_2_K_*T*_	0.700	0.825 (0.0009)	0.800	0.850 (0.0007)	0.900	0.900 (0.0128)
PK_*T*_	0.725	0.700 (0.0006)	0.800	0.725 (0.0004)	0.750	0.750 (0.0019)
						
	Potato					
	
	GPR		PIR		PM	
			
K_*T*_	0.525	0.500 (0.0065)	0.625	0.550 (0.0034)	0.475	0.475 (0.0082)
Q_1_K_*T*_	0.600	0.600 (0.0054)	0.625	0.550 (0.0033)	0.475	0.525 (0.0086)
Q_2_K_*T*_	0.600	0.600 (0.0091)	0.625	0.500 (0.0010)	0.625	0.525 (0.0031)
PK_*T*_	0.575	0.550 (0.0121)	0.625	0.550 (0.0048)	0.475	0.475 (0.0153)
						
	Sugar beet					
	
	AN		BY		CSY	
			
K_*T*_	0.575	0.325 (0.0022)	0.475	0.300 (0.0022)	0.475	0.350 (0.0012)
Q_1_K_*T*_	0.575	0.325 (0.0023)	0.450	0.300 (0.0059)	0.475	0.375 (0.0006)
Q_2_K_*T*_	0.475	0.325 (0.0029)	0.475	0.275 (0.0021)	0.575	0.350 (0.0009)
PK_*T*_	0.475	0.300 (0.0019)	0.350	0.300 (0.0034)	0.475	0.350 (0.0009)
						
	Maize					
	
	EH		ED		DPS	
			
K_*T*_	0.575	0.450 (0.0002)	0.575	0.575 (0.0001)	0.600	0.575 (0.0002)
Q_1_K_*T*_	0.575	0.500 (0.0001)	0.725	0.575 (0.0003)	0.600	0.575 (0.0001)
Q_2_K_*T*_	0.875	0.475 (0.0003)	0.725	0.525 (0.0001)	0.600	0.525 (0.0001)
PK_*T*_	0.875	0.475 (0.0002)	0.725	0.525 (0.0001)	0.600	0.600 (0.0001)
						
	Arabidopsis					
	
	FLC		FRI		LDV	
			
K_*T*_	0.875	0.875 (0.0004)	0.825	0.975 (0.0007)	0.875	0.900 (0.0034)
Q_1_K_*T*_	0.875	0.975 (0.0023)	0.875	0.800 (0.0028)	0.875	0.900 (0.0020)
Q_2_K_*T*_	0.875	0.950 (0.0006)	0.875	0.800 (0.0018)	0.875	0.900 (0.0017)
PK_*T*_	0.875	0.775 (0.0015)	0.925	0.925 (0.0025)	0.900	0.900 (0.0011)

For the latter measure, the observed mean of squared differences are given in parentheses. For abbreviations of the analyzed traits see Table 2. For a detailed definition of the statistical models and description of the different methods see Materials and Methods.

The 95% quantile of differences in MSD calculated for the five pairs of association methods Q_1_K/ANOVA, Q_1_K/K, Q_1_K/Q_2_K, Q_1_K/PK, and Q_1_K/$Q_{1}{\text{K}}_{T_{opt}}$ was highest for potato and ranged from 0.0041 to 0.0114 (Additional file 5). For Arabidopsis, the 95% quantile of differences in MSD was lowest and varied from 0.0001 to 0.0004.

**Figure 2:**
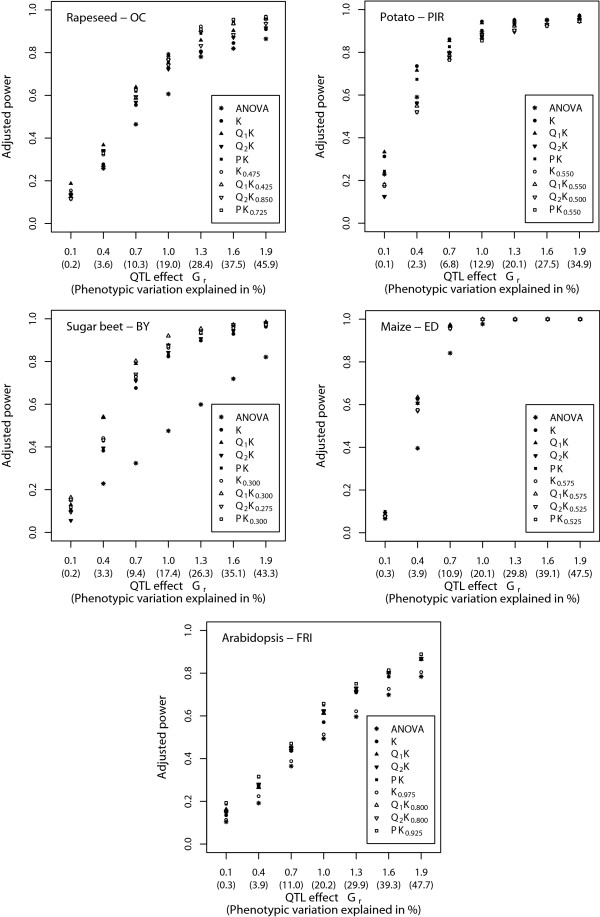
**Adjusted power to detect quantitative trait loci (QTL) for the nine different association mapping methods depending on the size of the QTL effect *G*_*r*_**. The percentage of phenotypic variation explained by a QTL was calculated for the average allele frequency (see Table 2). For each of the five plant species, the result of the trait with medium genetic complexity is presented.

The slopes of the power curve were flat for small as well as large genetic effects, whereas for genetic effects of medium size the slope was steep (Fig. 2). For most traits under consideration, the adjusted power of the $Q_{1}{\text{K}}_{T_{opt}}$, $Q_{2}{\text{K}}_{T_{opt}}$, and ${\text{PK}}_{T_{opt}}$ methods was slightly higher across all examined sizes of genetic effects than those of the Q_1_K, Q_2_K, and PK methods. In comparison with the other association mapping methods, the ANOVA method showed the lowest adjusted power to detect QTL across all examined sizes of genetic effects for all traits and plant species except potato (PIR).

The Pearson correlation coefficient between the observed *P *values of all examined association mapping methods ranged from -0.05 to 0.99 (Additional file 6)

## Discussion

### Assumptions underlying the comparison of association mapping approaches using empirical data sets

Simulation of data sets mimicing the population structure and familial relatedness of empirical data sets is hardly possible. However, only with such data sets a reliable assessment of the performance of different association mapping approaches is possible. Therefore, our study was based on empirical data sets.

Investigations on the type I error rate and on the adjusted power to detect QTL of association mapping approaches using empirical data sets require that the examined marker loci are unlinked to polymorphisms controlling the expression of the trait under consideration. In the present study, this assumption seems to be reasonable as for the five plant species examined the available marker density was considerably lower than that required for genome-wide association mapping. Similarly to other studies comparing association mapping approaches based on empirical data [15,17], however, we cannot rule out the possibility that some markers might be linked to functional polymorphisms of the traits under consideration.

In accordance with previous studies [15,17], we used the same markers for estimation of population structure as well as familial relatedness as were used for calculating the MSD between observed and expected *P *values. Theoretical considerations suggest that MSD values calculated in this way might underestimate the MSD values for markers which are not included in the estimation of population structure and familial relatedness such as markers in candidate genes. However, our computer simulations on the Arabidopsis dataset, in which the half of the available markers were used for estimation of population structure and familial relatedness and the remaining markers for calculation of the MSD values, suggested that this underestimation is negligible (data not shown). This result indicates that association mapping methods, for which we observed MSD values close to zero, will also adhere to the nominal *α *level in empirical association mapping experiments.

Our power simulations assumed a QTL allele which is in complete LD with one marker allele. This assumption allows the comparison of results from various plant species irrespective of the available number of markers. However, it maximizes the power for QTL detection. In most empirical studies no markers are available which are in complete LD with the QTL. Therefore, for such studies, a lower power for QTL detection is expected depending on the extent of LD between marker and QTL. A further factor influencing the detection of the QTL of interest, which was neglected in our power simulations, are additional QTL that are linked to the QTL of interest. Incomplete LD between marker and QTL as well as additional linked QTL are expected to alter the power of QTL detection in all association mapping methods to a similar extent. Therefore, no influence on our conclusions regarding the ranking of various methods for association mapping is expected with respect to the assumptions made in our power simulations.

### Comparison of various association mapping approaches

#### ANOVA approach

A frequently used method for association mapping in a plant genetics context is the ANOVA approach [10]. This approach was therefore used in our study as reference method. Under the assumption that the random marker loci in our study are unlinked to the polymorphisms controlling the expression of the traits under consideration, association mapping methods that adhere to the nominal *α *level show a uniform distribution of *P *values, *i.e*., a MSD value close to zero. With the exception of the normalized gene expression data of the FRI gene in Arabidopsis, we observed a non-uniform distribution of *P *values in the ANOVA approach of all traits (Table 3). This finding is in accordance with the results of previous studies [15,17,20] and indicates that the ANOVA approach is inappropriate for association mapping in the examined plant species, because the resulting proportion of spurious marker-phenotype associations is considerably higher than the nominal type I error rate.

#### QK approach

The recently proposed QK mixed-model association mapping method promises to correct for multiple levels of relatedness [15]. The MSD between observed and expected *P *values found for the Q_1_K and Q_2_K methods of all examined traits was considerably lower than that observed for the ANOVA approach (Table 3). Furthermore, this difference in MSD values was considerably larger than the 95% quantile observed based on the computer simulations (Additional file 5). These findings suggest the advantage of the Q_1_K and Q_2_K methods over the ANOVA method for association mapping not only in maize and Arabidopsis for which similar results were previously reported [15,17] but also in rapeseed, potato, and sugar beet.

For estimation of the number of sub-populations using STRUCTURE [29], Δ*K*, an ad hoc criterion related to the second order rate of change in the log likelihood of data, was proposed [31]. In other studies, the number of sub-populations *z*+1 was chosen in such a way that a further increase in *z *did not considerably improve the log likelihood of data [35]. We used these two criteria to estimate the number of sub-populations for the **Q**_1 _and **Q**_2 _matrices.

For some traits, we observed a smaller MSD value for the Q_1_K than for the Q_2_K method, whereas the opposite was true for the other traits (Table 3). Furthermore, with few exceptions, these differences were smaller than the corresponding 95% quantiles observed in our computer simulations on the correlated beta-distribution (Additional file 5). These findings demonstrate that the association mapping models based on the two population structure matrices, **Q**_1 _and **Q**_2_, are equally appropriate for association mapping with respect to (i) adherence to the nominal *α *level as well as (ii) the adjusted power for QTL detection.

Despite promising results for the Q_1_K and Q_2_K association mapping approaches, these methods have several drawbacks, as previously discussed [20]. Therefore, we examined two association mapping methods which were not based on the population structure matrix from STRUCTURE. For the PK mixed-model association mapping approach, the **Q**_1 _or **Q**_2 _matrix from STRUCTURE was replaced by a matrix comprising *p *principal components (Table 1). In contrast, the K method was based on a mixed-model which does not include any *v*_*u *_effects.

#### PK approach

The MSD between observed and expected *P *values, which was found for this method, was similar to those observed for the Q_1_K and Q_2_K methods (Table 3). Furthermore, all three methods yielded a similar adjusted power of QTL detection across the examined plant species (Fig. 2). These findings were in accordance with those of previous studies [17,20], suggesting that the PK approach is a promising alternative to the Q_1_K and Q_2_K methods.

#### K approach

For the K approach, we observed for most examined traits a higher MSD value than for the mixed-model methods Q_1_K, Q_2_K, and PK. The opposite result was observed with respect to the adjusted power of QTL detection (Fig. 2).

These results indicated that the K approach was less appropriate for association mapping than the approaches based on the integration of fixed effects in the statistical model. This conclusion may be explained by the fact that the software package SPAGeDi [28] used for calculation of the kinship coefficients assumes that random pairs of individuals of the germplasm set under consideration are unrelated and assigns them a kinship coefficient of 0. This definition of unrelated individuals results in a kinship matrix for which a large number of pair-wise kinship estimates are negative. It was proposed to replace these negative values by 0, because such pairs of individuals are less related than random pairs of individuals [15]. This approach, however, ignores information on the structure of unrelated individuals, which was captured in the kinship matrix, and consequently necessitates the inclusion of fixed effects in the mixed-model. Therefore, we examined mixed-model association mapping approaches which are based on **K **matrices calculated for different thresholds *T *[20].

#### Approaches based on K matrices calculated for different values of *T*

The values of *T*_*opt *_calculated for the current data sets using the REML approach, which might also be used to infer the probability of identity by descent for genotypes with no pedigree information available, were not always identical with those identified based on the MSD profiles (Table 4). Across all plant species, traits, and association mapping methods, however, the correlation between the *T*_*opt *_value identified based on both approaches was 0.83 (Additional file 4). This result suggested that for association mapping approaches the T_*opt *_value might be identified using the REML approach because it is associated with a lower computational load. The REML-based deviance, used to estimate *T*_*opt*_, however, can only be compared among models which are based on the same set of fixed effects. Therefore, we used the MSD between observed and expected *P *values for comparison of the Q_1_K_*T*_, Q_2_K_*T*_, PK_*T*_, and K_*T *_method and furthermore used the *T*_*opt *_values identified based on this criterion.

The MSD values observed for the association mapping approaches based on the *T*_*opt *_value, were considerably lower than that of the corresponding association mapping approaches based on the **K **matrix from SPAGeDi, for all examined plant species and traits (Table 4). Furthermore, the adjusted power observed for the former approaches was for most examined traits higher than that observed for the latter approaches. These findings suggest that methods based on a kinship matrix calculated for the *T*_*opt *_value are more appropriate for association mapping than the corresponding association mapping approaches which are based on the **K **matrix from SPAGeDi. Nevertheless, the MSD values observed for the association mapping methods, which include fixed effects such as the $Q_{1}{\text{K}}_{T_{opt}}$, $Q_{2}{\text{K}}_{T_{opt}}$, or ${\text{PK}}_{T_{opt}}$, were lower than that of the ${\text{K}}_{T_{opt}}$. Therefore, in our study the $Q_{1}{\text{K}}_{T_{opt}}$, $Q_{2}{\text{K}}_{T_{opt}}$ or ${\text{PK}}_{T_{opt}}$ are the most appropriate methods for association mapping.

### Comparison of the properties of association mapping approaches among plant species and traits

#### MSD values

The MSD values observed for potato and sugar beet across all association mapping methods were considerably higher than those for maize and Arabidopsis, whereas those for rapeseed were of medium size (Table 3). This may be due to the low number of random molecular markers available in our study for potato, sugar beet, and rapeseed. Thereby, not very precise estimation of population structure is possible which in turn increases the MSD values.

To examine this issue in more detail, random markers were selected in replicated simulation runs from maize and Arabidopsis linkage maps in such a way that the total number of alleles of the selected markers corresponds to those observed for the other three species. All association mapping methods were then run with these markers. Our results (data not shown) suggested that the low number of random molecular markers for potato, sugar beet, and rape seed only partially explains the observed differences in MSD values.

Another factor that explains the observed difference in MSD values among the plant species is the difference in the extent of population structure and relatedness present in the examined genetic materials. This difference in population structure and relatedness may partly be due to the fact that the entries of the examined plant species differ in their origin. While the Arabidopsis entries were selected from natural populations, the entries of the other four plant species were chosen from plant breeding programs. Because entries selected from plant breeding programs have a complex ancestry, the extent of population structure and relatedness in such germplasm sets is expected to be higher than in germplasm sets consisting of entries selected from natural populations.

In addition, the difference in the extent of population structure and relatedness between rapeseed, potato, sugar beet, and maize can be explained by the different sampling strategies underlying the examined genetic materials. The entries of the maize data set represent world-wide genetic diversity, whereas the genetic materials of rapeseed, potato, and sugar beet were sampled from commercial plant breeding programs. Theoretical considerations suggest that this increases the probability of including partially related entries.

Furthermore, the difference in the extent of population structure and relatedness between rapeseed, potato, sugar beet, and maize may partly be due to the different reproduction systems and types of varieties bred in a particular crop. For entries from hybrid breeding programs [11] such as sugarbeet and maize, distinct sub-poulations are expected. In contrast, when line or clonal varieties are bred, as in the case of rapeseed and potato, no distinct sub-populations are expected to develop as population structure is disregarded when choosing the parents of a cross. Nevertheless, this procedure is expected to generate diverse levels of familial relatedness [36].

#### Adjusted power for QTL detection

Across all examined statistical methods for association mapping, considerable differences in the adjusted power for QTL detection were observed for the five examined plant species (Fig. 2). The adjusted power is influenced by (i) the size of the QTL effect *G*_*r*_, (ii) the extent of LD between marker allele and QTL allele, (iii) the number of entries *n*, (iv) the QTL allele frequency, and (v) the heritability of the trait under consideration. Our power simulations assumed the same QTL effects for all plant species and a QTL allele which is in complete LD with one marker allele. These two factors cannot contribute to the observed difference in adjusted power for QTL detection among the examined plant species.

High adjusted power for the maize data set with its high number of entries and a low adjusted power for the Arabidopsis data set with a low number of entries indicated that differences in the number of entries *n *have a large influence on the observed differences in adjusted power among the examined plant species. This explanation is supported by results of previous studies [37]. In contrast, the small difference in adjusted power for QTL detection between sugar beet and potato data sets, which comprised a similar number of entries but differed in their average allele frequency, suggested that variation in this factor caused only small differences in the adjusted power.

In our study, heritability estimates were only available for two plant species and, thus, no inferences can be made about the contribution of this factor to differences in the adjusted power for QTL detection. However, results from previous studies suggested that increasing heritability has the potential to considerably increase the power for QTL detection [14].

#### T_*opt*_

The optimum T values identified in our study differed considerably among the various plant species (Table 4). This finding may be due to the difference in the extent of population structure and familial relatedness among the examined plant species as described above. The influence of population structure and familial relatedness on the optimum *T *value can be explained by the fact that lower values for *T *reduce the number of negative pair-wise kinship estimates in the kinship matrix **K**_*T*_. Thereby the use of information concerning the structure of unrelated individuals, which was comprised in the kinship matrix **K**_*T*_, is improved and decreases the MSD values.

In comparison with the large differences among the optimum *T *values identified for different plant species, differences in the optimum *T *values for different traits of the same species were only small (Table 4). This finding might be explained by the fact that differences in the optimum *T *values identified for different traits of the same plant species can only be due to differences in the extent of population structure and relatedness for the traits under consideration generated by natural or artificial selection. Therefore, one optimum *T *value might be calculated across all traits of one species to improve the precision of this value. However, this requires further research on the standard error of the optimum *T *values.

## Conclusion

Our study suggests that the QK method [15] is not only appropriate for association mapping in humans, maize, and Arabidopsis but also in rapeseed, potato, and sugar beet. Furthermore, our results indicate that the estimation of the number of sub-populations based on the two criteria, Δ*K *and SBC, results in different numbers of sub-populations. Nevertheless, the association mapping models which are based on these two population structure matrices are equally appropriate with respect to adherence to the nominal *α *level as well as the adjusted power for QTL detection. Furthermore, we recommend replacing the **K **matrix of the Q_1_K, Q_2_K, and PK approach by a **K**_*T *_matrix, which is based on a REML estimate of the conditional probability that two inbreds carry alleles at the same locus which are identical in state but not identical by descent and, thus, increase the adherence to the nominal *α *level. Finally, we showed that the *T*_*opt *_value estimated in this way differs considerably among the five plant species but only a little for the different traits within species.

## Abbreviations

AN: amino nitrogen; BY: beet yield; CSY: corrected sugar yield; ED: ear diameter; EH: ear height; FLC: *FLOWERING LOCUS C*; FRI: *FRIGIDA*; GPR: *Globodera pallida *resistance; LD: linkage disequilibrium; LDV: long day conditions with vernalisation treatment; MSD: mean of squared difference; OC: oil content; OY: oil yield; PIR: *Phytophthora infestans *resistance; PM: plant maturity; QTL: quantitative trait locus; REML: restricted maximum likelihood; SBC: Schwarz Bayesian criterion; TKW: thousand kernel weight.

## Authors' contributions

BS designed the project and analyzed the data. BS and AEM wrote the manuscript.

## Supplementary Material

Additional file 1 **Plant materials, phenotypic data, and molecular markers description:** Description of the plant materials, phenotypic data, and molecular markers used for the study. Click here for file

Additional file 2 **Phenotypic data analyses.** Description of the statistical analyses of the phenotypic data. Click here for file

Additional file 3 **Comparison of four different mixed-model association mapping methods.** Mean of squared differences between observed and expected *P *values for four different mixed-model association mapping methods depending on the threshold *T*. Click here for file

Additional file 4 **Comparison of two methods for estimation of the threshold *T*.** Optimum values for threshold *T *identified based on mean of squared differences between observed and expected *P *values plotted versus optimum *T *values identified based on deviance for the four mixed-model association mapping methods of the five plant species and three traits. Click here for file

Additional file 5 **Difference in mean square differences between pairs of association mapping methods expected purely by chance.** Ninety-five % quantile of the difference of the mean square differences between observed and expected *P *values for five pairs of association mapping approaches determined based on a bivariate beta-distribution. Click here for file

Additional file 6 **Comparison of the results of various association mapping methods.** Pearson correlation coefficient between the observed *P *values for various association mapping methods. Click here for file
