# Supplementary material for: Comparison of mixed-model approaches for association mapping in rapeseed, potato, sugar beet, maize, and Arabidopsis
Source: BMC Genomics. 2009 Feb 27;10:94. doi: 10.1186/1471-2164-10-94 (PMC2676307; doi:10.1186/1471-2164-10-94)
Supplement: Additional file 1 — Plant materials, phenotypic data, and molecular markers description: Description of the plant materials, phenotypic data, and molecular markers used for the study. [file 1471-2164-10-94-S1.pdf]

ADDITIONAL FILE 1

Plant materials, phenotypic data, and molecular markers

Rapeseed (*Brassica napus* L.): We analyzed a total of $n = 136$ rapeseed inbreds proprietary to Norddeutsche Pflanzenzucht Hans-Georg Lembke KG (Holtsee, Germany). In 2004, all entries were evaluated in a series of field trials. All trials included a set of three common checks and were conducted at Hohenlieth and Malchow (Germany). The experimental design of each trial was a 6×6 lattice design with one replicate at each location. Data were recorded for thousand kernel weight (TKW; g), oil content (OC; %), and oil yield (OY; t/ha). The 136 entries were fingerprinted with $m = 59$ genome-wide distributed simple sequence repeat markers by Saaten-Union Resistenzlabor GmbH (Hovedissen, Germany) following standard protocols.

Potato (*Solanum tuberosum* L.): Our study was based on the phenotypic and genotypic data evaluated earlier [21]. In short, the breeding companies Böhm-Nordkartoffel Agrarproduktion OHG (Lüneburg, Germany) and Saka-Ragis Pflanzenzucht GbR (Windeby, Germany) selected 86 and 90 tetraploid potato clones from their breeding programs, respectively. From 2004 to 2006, both breeding companies evaluated their entries together with a set of eight common potato clones in pot experiments for *Globodera pallida* St. resistance (GPR) [22]. Our statistical analyses were based on the square root of the number of visible nematode cysts. In addition, in the same period, each breeding company grew 12 plants of each of their entries and the common potato clones in each of two replicates at one location. These plots were inoculated with *Phytophthora infestans* (Mont.)

de Bary and disease progress was monitored every 3-4 days using a 1-9 scale (1 = 0% infection, 9 > 90% infection). The area under the disease progress curve [23] calculated from these data was used as measure for *P. infestans* resistance (PIR). Furthermore, at each location, plant maturity (PM) was evaluated in uninfected plants grown under standard phytosanitary regimes, using a 1 to 9 scale (1 = very early, 9 = very late). The $n = 184$ clones and common entries were fingerprinted with $m = 31$ genome-wide distributed simple sequence repeat markers [21] by the potato genome analysis group of the Max Planck Institute for Plant Breeding Research (Cologne, Germany). For 21 markers the allele dosage was scored based on relative band intensities.

Sugar beet (*Beta vulgaris* L.): We analyzed a total of $n = 178$ sugar beet inbreds of the pollen parent heterotic pool, proprietary to KWS SAAT AG (Einbeck, Germany). The test-cross progenies of these entries with an inbred of the seed parent heterotic pool were evaluated in a series of plant breeding trials. All trials included a set of eight common checks and were conducted at six locations. The experimental design of each trial was a 10×10 lattice design with two replicates at each location. Data were recorded for amino nitrogen (AN) [24], beet yield (BY), and corrected sugar yield (CSY) [25] in % of the mean performance of the checks. All entries were fingerprinted with 59 simple sequence repeat markers and 41 single nucleotide polymorphism markers ($m = 100$), both randomly distributed across the sugar beet genome. The fingerprinting was conducted by KWS SAAT AG following standard protocols.

Maize (*Zea mays* L.): Our study was based on the phenotypic and genotypic data analyzed earlier [15]. The $n = 277$ maize inbreds represented world-wide genetic diversity. Field tests were conducted at Clayton (NC; USA) and Home-

stead (FL; USA) in 2002. We analyzed ear height (EH; cm), ear diameter (ED; cm), and days to pollen shed (DPS). For all inbreds, genotypic data of $m = 553$ genome-wide distributed single nucleotide polymorphism markers was available.

***Arabidopsis thaliana* L.:** Our study was based on the $n = 95$ *Arabidopsis thaliana* L. inbreds for which phenotypic information was available [17]. These inbreds represent world-wide genetic diversity of Arabidopsis. We examined the normalized gene expression of *FLOWERING LOCUS C* (FLC) and *FRIGIDA* (FRI) as well as the number of days from germination to first opening of flowers under long day conditions with vernalisation treatment (LDV). For these inbreds, resequencing data of $m = 876$ genome-wide distributed short fragments was available [26]. To reduce the computational load, we used only the central single nucleotide polymorphism marker of each fragment.
