# Supplementary material for: Comparison of mixed-model approaches for association mapping in rapeseed, potato, sugar beet, maize, and Arabidopsis
Source: BMC Genomics. 2009 Feb 27;10:94. doi: 10.1186/1471-2164-10-94 (PMC2676307; doi:10.1186/1471-2164-10-94)
Supplement: Additional file 2 — Phenotypic data analyses. Description of the statistical analyses of the phenotypic data. [file 1471-2164-10-94-S2.pdf]

ADDITIONAL FILE 2

Phenotypic data analyses: The phenotypic data for rapeseed were analyzed based on the statistical model:

$$y_{ijko} = \mu + c_i + d_i g_i + l_j + t_{kj} + b_{ojk} + e_{ijko} \quad ,$$

where y_{ijko} was the phenotypic observation for the ith individual at the jth location in the oth incomplete block of the kth trial, μ an intercept term, c_i a factor with a single level for each check and a single level for all entries, d_i an indicator variable with $d_i = 0$ for checks and $d_i = 1$ for entries, g_i the genotypic effect of the ith entry, l_j the effect of the jth location, t_{kj} the effect of the kth trial at the jth location, b_{ojk} the effect of the oth incomplete block of the kth trial at the jth location, and e_{ijko} the residual. For details regarding the use of dummy coding for separating checks and entries see [38]. Because trials were only performed with one replicate per location, no inferences can be made about genotype×location interactions. For estimation of variance components, c_i and l_j were considered as fixed and all other effects as random.

For estimating entry means, we regarded c_i, $d_i g_i$, and l_j as fixed and all other effects as random. Over all trials, an adjusted entry mean M_i was calculated for each of the n entries as:

$$M_i = \hat{\mu} + \widehat{g_i} \quad ,$$

where $\hat{\mu}$ and $\widehat{g_i}$ denote the generalized least square estimates of μ and g_i, respectively.

For the phenotypic data analyses of potato, each year-location combination

was treated as an environment and entry means for each environment were used for the analyses:

$$y_{ij} = \mu + g_i + l_j + e_{ij} \quad ,$$

where y_{ij} was the entry mean for the ith clone in the jth environment, μ an intercept term, g_i the genotypic effect of the ith clone, l_j the effect of the jth environment, and e_{ij} the residual. Because environments comprised two selected locations, the environmental effects l_j were regarded as fixed. For estimation of variance components, g_i was considered as random, whereas for estimating entry means, g_i was considered as fixed. Over all environments, an adjusted entry mean M_i was calculated for each clone as:

$$M_i = \hat{\mu} + \widehat{g_i} \quad .$$

For rapeseed and potato, heritability on an entry mean basis h^2 was calculated as:

$$h^2 = \frac{\sigma_g^2}{\sigma_g^2 + \bar{w}/2} \quad ,$$

where σ_g^2 was the genotypic variance and $\bar{w}$ the mean variance of the difference between two adjusted entry means [39].

For sugar beet, maize, and Arabidopsis, we used the entry means M_i for the various association mapping approaches.
