# Supplementary material for: Comparison of mixed-model approaches for association mapping in rapeseed, potato, sugar beet, maize, and Arabidopsis
Source: BMC Genomics. 2009 Feb 27;10:94. doi: 10.1186/1471-2164-10-94 (PMC2676307; doi:10.1186/1471-2164-10-94)
Supplement: Additional file 3 — Comparison of four different mixed-model association mapping methods. Mean of squared differences between observed and expected P values for four different mixed-model association mapping methods depending on the threshold T. [file 1471-2164-10-94-S3.pdf]

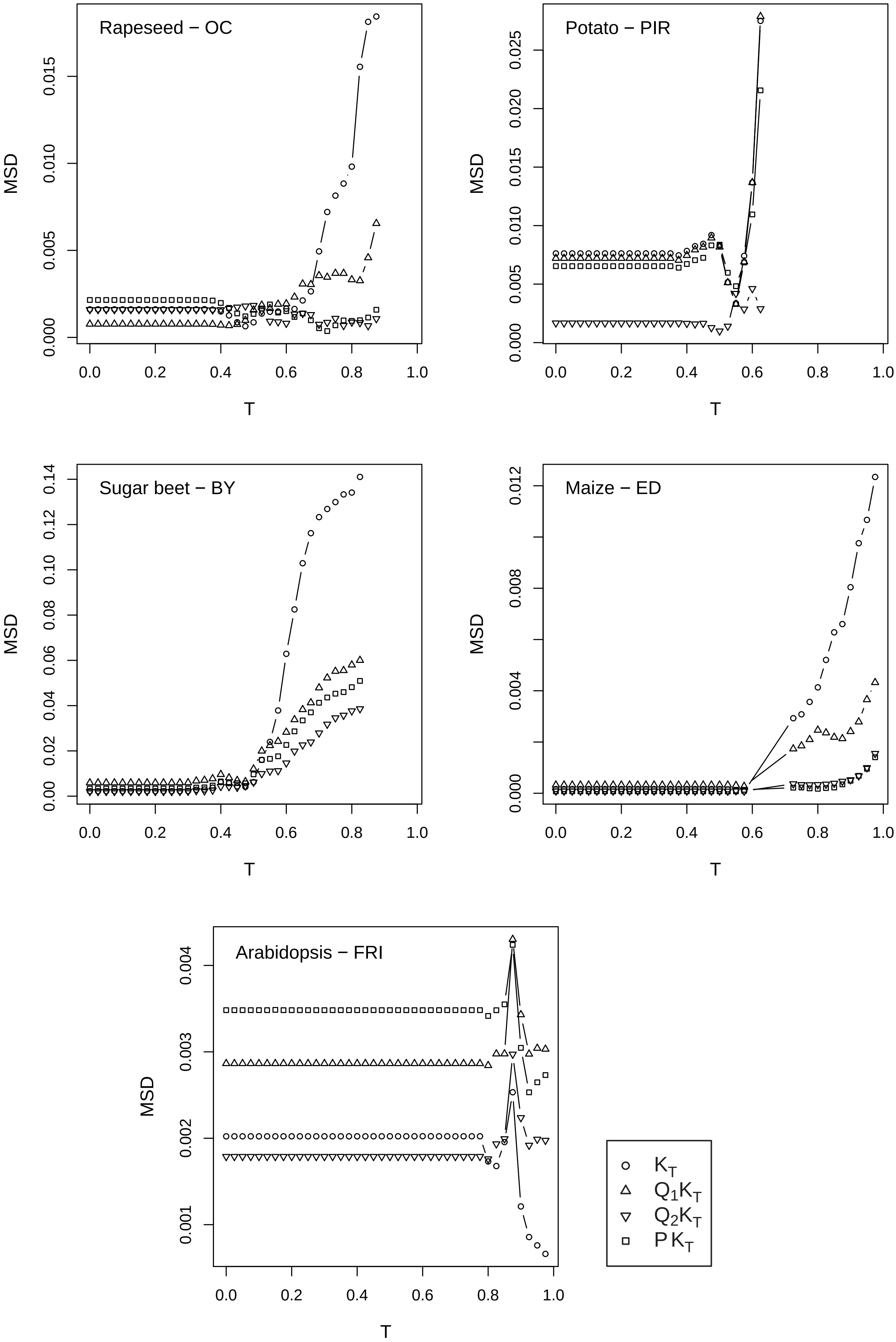

Additional file 3. Mean of squared differences (MSD) between observed and expected P values for four different mixed-model association mapping methods depending on threshold T. For each of the five plant species, the result of the trait with medium genetic complexity is presented. The incomplete profiles for rapeseed, potato, and sugarbeet are due to convergence problems for the corresponding T values.
