# Supplementary material for: Comparison of mixed-model approaches for association mapping in rapeseed, potato, sugar beet, maize, and Arabidopsis
Source: BMC Genomics. 2009 Feb 27;10:94. doi: 10.1186/1471-2164-10-94 (PMC2676307; doi:10.1186/1471-2164-10-94)
Supplement: Additional file 4 — Comparison of two methods for estimation of the threshold T. Optimum values for threshold T identified based on mean of squared differences between observed and expected P values plotted versus optimum T values identified based on deviance for the four mixed-model association mapping methods of the five plant species and three traits. [file 1471-2164-10-94-S4.pdf]

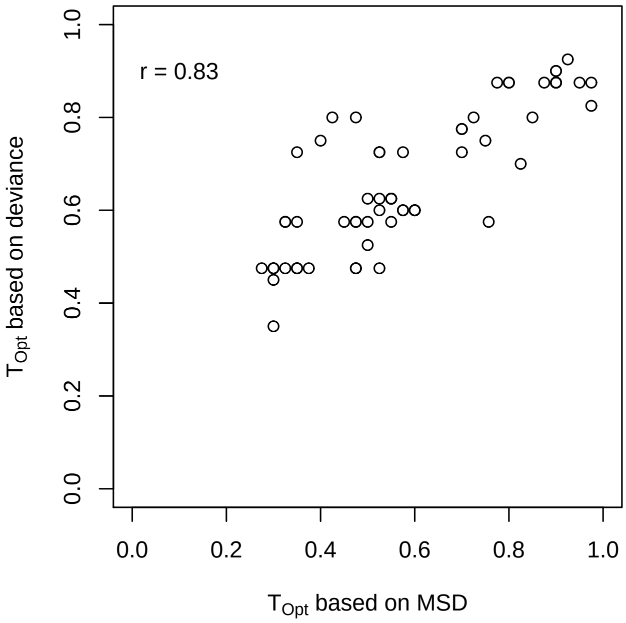

Additional file 4. Optimum values for threshold T identified based on mean of squared differences (MSD) between observed and expected P values plotted versus optimum T values identified based on deviance for the four mixed-model association mapping methods of the five plant species and three traits.
