# Supplementary material for: Comparison of mixed-model approaches for association mapping in rapeseed, potato, sugar beet, maize, and Arabidopsis
Source: BMC Genomics. 2009 Feb 27;10:94. doi: 10.1186/1471-2164-10-94 (PMC2676307; doi:10.1186/1471-2164-10-94)
Supplement: Additional file 5 — Difference in mean square differences between pairs of association mapping methods expected purely by chance. Ninety-five % quantile of the difference of the mean square differences between observed and expected P values for five pairs of association mapping approaches determined based on a bivariate beta-distribution. [file 1471-2164-10-94-S5.pdf]

Additional file 5. Ninety-five % quantile of the difference of the mean square differences (MSD) between observed and expected P values for five pairs of association mapping approaches determined based on a bivariate beta-distribution. For abbreviations of the analyzed traits see Table 2. For a detailed definition of the statistical models and description of the different methods see Materials and Methods.

Trait	95 % quantile of the difference in MSD				
	Q_1K				
	ANOVA	K	Q_2K	PK	$Q_1K_{T_{opt}}$
	Rapeseed				
TKW	0.0057	0.0049	0.0050	0.0031	0.0033
OC	0.0051	0.0042	0.0056	0.0049	0.0028
OY	0.0059	0.0049	0.0050	0.0051	0.0033
	Potato				
GPR	0.0114	0.0097	0.0093	0.0051	0.0041
PIR	0.0075	0.0022	0.0106	0.0066	0.0065
PM	0.0084	0.0059	0.0101	0.0094	0.0049
	Sugar beet				
AN	0.0034	0.0021	0.0014	0.0016	0.0023
BY	0.0034	0.0034	0.0029	0.0021	0.0017
CSY	0.0034	0.0029	0.0024	0.0020	0.0018
	Maize				
EH	0.0006	0.0003	0.0003	0.0004	0.0003
ED	0.0006	0.0003	0.0003	0.0004	0.0004
DPS	0.0006	0.0005	0.0004	0.0004	0.0003
	Arabidopsis				
FLC	0.0003	0.0003	0.0002	0.0003	0.0002
FRI	0.0003	0.0003	0.0001	0.0002	0.0001
LDV	0.0004	0.0003	0.0001	0.0003	0.0002
