# Supplementary material for: Comparison of mixed-model approaches for association mapping in rapeseed, potato, sugar beet, maize, and Arabidopsis
Source: BMC Genomics. 2009 Feb 27;10:94. doi: 10.1186/1471-2164-10-94 (PMC2676307; doi:10.1186/1471-2164-10-94)
Supplement: Additional file 6 — Comparison of the results of various association mapping methods. Pearson correlation coefficient between the observed P values for various association mapping methods. [file 1471-2164-10-94-S6.pdf]

Additional file 6. Pearson correlation coefficient between the observed *P* values for various association mapping methods. For each of the five plant species, the result of the trait with medium genetic complexity is presented. For abbreviations of the analyzed traits see Table 2. For a detailed definition of the statistical models and description of the different methods see Materials and Methods.

	Rapeseed – OC								
ANOVA	1.00	0.81	0.50	0.06	0.32	0.53	0.49	-0.02	0.16
K	0.81	1.00	0.70	0.24	0.44	0.78	0.71	0.19	0.33
Q_1K	0.50	0.70	1.00	0.27	0.54	0.85	0.89	0.24	0.47
Q_2K	0.06	0.24	0.27	1.00	0.62	0.31	0.34	0.90	0.57
PK	0.32	0.44	0.54	0.62	1.00	0.64	0.65	0.67	0.88
$K_{0.475}$	0.53	0.78	0.85	0.31	0.64	1.00	0.97	0.29	0.58
$Q_1K_{0.425}$	0.49	0.71	0.89	0.34	0.65	0.97	1.00	0.31	0.59
$Q_2K_{0.850}$	-0.02	0.19	0.24	0.90	0.67	0.29	0.31	1.00	0.67
$PK_{0.725}$	0.16	0.33	0.47	0.57	0.88	0.58	0.59	0.67	1.00
	Potato – PIR								
ANOVA	1.00	0.77	0.77	-0.05	0.64	0.58	0.58	0.06	0.44
K	0.77	1.00	0.98	0.31	0.84	0.83	0.83	0.42	0.73
Q_1K	0.77	0.98	1.00	0.31	0.83	0.83	0.83	0.40	0.75
Q_2K	-0.05	0.31	0.31	1.00	0.49	0.34	0.34	0.83	0.48
PK	0.64	0.84	0.83	0.49	1.00	0.85	0.85	0.54	0.85
$K_{0.550}$	0.58	0.83	0.83	0.34	0.85	1.00	1.00	0.51	0.95
$Q_1K_{0.550}$	0.58	0.83	0.83	0.34	0.85	1.00	1.00	0.50	0.95
$Q_2K_{0.550}$	0.06	0.42	0.40	0.83	0.54	0.51	0.50	1.00	0.62
$PK_{0.550}$	0.44	0.73	0.75	0.48	0.85	0.95	0.95	0.62	1.00
	Sugar beet – BY								
ANOVA	1.00	0.45	0.11	0.10	0.17	0.21	0.04	0.11	0.14
K	0.45	1.00	0.57	0.55	0.60	0.74	0.49	0.53	0.56
Q_1K	0.11	0.57	1.00	0.72	0.83	0.66	0.86	0.64	0.71
Q_2K	0.10	0.55	0.72	1.00	0.69	0.58	0.67	0.88	0.56
PK	0.17	0.60	0.83	0.69	1.00	0.77	0.82	0.61	0.86
$K_{0.300}$	0.21	0.74	0.66	0.58	0.77	1.00	0.82	0.64	0.89
$Q_1K_{0.300}$	0.04	0.49	0.86	0.67	0.82	0.82	1.00	0.70	0.89
$Q_2K_{0.275}$	0.11	0.53	0.64	0.88	0.61	0.64	0.70	1.00	0.65
$PK_{0.300}$	0.14	0.56	0.71	0.56	0.86	0.89	0.89	0.65	1.00
	Maize – ED								
ANOVA	1.00	0.32	0.19	0.14	0.16	0.40	0.22	0.19	0.21
K	0.32	1.00	0.89	0.83	0.81	0.79	0.71	0.68	0.62
Q_1K	0.19	0.89	1.00	0.90	0.84	0.66	0.81	0.75	0.65
Q_2K	0.14	0.83	0.90	1.00	0.83	0.58	0.71	0.81	0.61
PK	0.16	0.81	0.84	0.83	1.00	0.68	0.72	0.69	0.77
$K_{0.575}$	0.40	0.79	0.66	0.58	0.68	1.00	0.81	0.69	0.77
$Q_1K_{0.575}$	0.22	0.71	0.81	0.71	0.72	0.81	1.00	0.83	0.82
$Q_2K_{0.525}$	0.19	0.68	0.75	0.81	0.69	0.69	0.83	1.00	0.76
$PK_{0.525}$	0.21	0.62	0.65	0.61	0.77	0.77	0.82	0.76	1.00
	Arabidopsis – FRI								
ANOVA	1.00	0.92	0.53	0.50	0.42	0.99	0.54	0.51	0.42
K	0.92	1.00	0.70	0.66	0.56	0.94	0.70	0.67	0.55
Q_1K	0.53	0.70	1.00	0.95	0.80	0.57	0.99	0.95	0.80
Q_2K	0.50	0.66	0.95	1.00	0.80	0.53	0.95	0.99	0.79
PK	0.42	0.56	0.80	0.80	1.00	0.45	0.83	0.82	0.99
$K_{0.975}$	0.99	0.94	0.57	0.53	0.45	1.00	0.58	0.54	0.45
$Q_1K_{0.800}$	0.54	0.70	0.99	0.95	0.83	0.58	1.00	0.95	0.82
$Q_2K_{0.800}$	0.51	0.67	0.95	0.99	0.82	0.54	0.95	1.00	0.81
$PK_{0.925}$	0.42	0.55	0.80	0.79	0.99	0.45	0.82	0.81	1.00
